# Social network inheritance and differentiation in wild baboons

**DOI:** 10.1098/rsos.230219

**Published:** 2023-05-24

**Authors:** Vittoria Roatti, Guy Cowlishaw, Elise Huchard, Alecia Carter

**Affiliations:** ^1^ Anthropology Department, University College London, London WC1E 6BT, UK; ^2^ Zoological Society of London, Institute of Zoology, London NW1 4RY, UK; ^3^ Institut des Sciences de l'Evolution de Montpellier, CNRS, University of Montpellier, 34095 Montpellier Cedex 5, France

**Keywords:** baboon, inheritance, differentiation, primate, social network, social learning

## Abstract

Immatures' social development may be fundamental to understand important biological processes, such as social information transmission through groups, that can vary with age and sex. Our aim was to determine how social networks change with age and differ between sexes in wild immature baboons, group-living primates that readily learn socially. Our results show that immature baboons inherited their mothers' networks and differentiated from them as they aged, increasing their association with partners of similar age and the same sex. Males were less bonded to their matriline and became more peripheral with age compared to females. Our results may pave the way to further studies testing a new hypothetical framework: in female-philopatric societies, social information transmission may be constrained at the matrilineal level by age- and sex-driven social clustering.

## 1. Introduction

For group-living species, social connections can affect fitness by influencing health and survival [1]. For example, strong social bonds enhance longevity, and both adult and offspring survival in wild baboons (*Papio* spp.) [2–6], killer whales (*Orcinus orca*) [7] and spotted hyenas (*Crocuta crocuta*) [8]. Yet despite the importance of an individuals’ social connectedness in many social species (but see eastern grey kangaroos *Macropus giganteus* [9]), the mechanism driving this relationship is unclear. An individual's position in the social network could affect a range of biological processes influencing fitness, such as the acquisition of information from knowledgeable group members (i.e. social learning) [10]. Social connections may be particularly important for immature age classes, who could benefit from social information more than adults due to their inexperience of the environment [11].

Social learning can be adaptive because it allows access to vital information without expending the time and energy needed for asocial learning [11,12] and can lead to variations in fitness [13,14]. For instance, older African elephant (*Loxodonta africana*) matriarchs have relatively greater conspecific call discrimination abilities, which can affect the behavioural responses of their family group and, consequently, enhance their reproductive success [13]. Social learning is closely tied to social connections: the number and characteristics of individuals' connections set an upper limit on social learning *opportunities* [15–18] and often predict information transmission. For example, socially central chacma baboons (*P. ursinus*) have better access to social information (e.g. about the location of food) because they have a larger number of social partners to provide that information compared to peripheral ones [19]. Recent studies have quantified social connections using social network analysis, which can account simultaneously for all the interactions occurring in a group, determining both an individual's direct and indirect connections in the network [20].

Social connections can be characterized through proximity (i.e. individuals are associating if they share similar spatial positions) or interactions (i.e. individuals are associating if they are observed interacting) [21]. Proximity may be important because individuals could acquire information more easily from others that are in visual contact. For example, spatial associations predict the discovery of foraging tasks by threespine sticklebacks (*Gasterosteous aculeatus*) [22] and experimental food patches in sympatric tits (Family Paridae) [23]. Alternatively, or concurrently, individuals may be more attentive to those with whom they share strong bonds. For instance, ring-tailed lemurs (*Lemur catta*) [24] and ravens (*Corvus corax*) [25] who are more central in an affiliation network are more likely to acquire information about the solution of a foraging task. Although our knowledge of social networks has increased dramatically in recent years [26], few studies have focused specifically on immature age classes, and little is known about how their social networks, and social learning opportunities, develop with age [15].

Understanding how social networks change during immatures' development may give important insights into which factors constrain or enhance group-level social learning. Immature individuals may both be more socially active than adults and rely more on social learning than asocial learning compared to adults [11,15]. A greater tendency to seek social opportunities was reported in juvenile mantled howler monkeys (*Alouatta palliata*) [27], rhesus macaques (*Macaca mulatta*) [28] and bottlenose dolphins (*Tursiops aduncus*) [29]. Because immature individuals may have social networks differentiated from those of adults (e.g. in Colombian spider monkeys *Ateles fusciceps rufiventris* [30]), they can play an important role in the group's network, to the point that their analytical exclusion changes the group's structure, as reported for olive baboons (*P. anubis*) [31]. In addition, juvenile individuals from various taxa show greater evidence of social learning than adults (e.g. chacma baboons [32], meerkats (*Suricata suricatta*) [33] and Australian skinks (*Eulamprus quoyii*) [34]). Despite the importance of immature animals' social connections for a group's ability to disseminate information [35], few studies have characterized changes in immature individuals' social bonds through time, in the wild, and in both sexes.

In this study, we draw on previous knowledge of primate social development to analyse immatures’ social networks in a wild population of a female-philopatric primate species, the chacma baboon. Primates offer wider opportunities to explore immatures' social connections compared to other taxa because they experience a relatively long period of juvenescence [36]. Because taxa that have a later age at first reproduction have been linked with higher social learning abilities [37], studying primate immatures’ social networks may set the bases for future studies on how these connections enhance or constrain social information transmission. Baboons are ideal models for this study because they live in large social groups [38], readily learn socially [19], and information transmission is predicted by both the proximity and grooming social networks [19]. Chacma baboons live in multi-male–multi-female societies [38]. Females are philopatric and form matrilines (i.e. social units including a mother and her offspring) within a linear and stable hierarchy, with offspring inheriting their mother's rank [39]. Males disperse at maturity (around 8 years old) [19]. Baboons learn socially about the location of new food patches [19] and may acquire information on what to eat by observing others while foraging and/or by inspecting the same food items others are feeding upon [16].

Juveniles’ networks may be shaped by the process of social inheritance and two individual traits: age and sex. Social inheritance suggests that offspring inherit their mother's social connections in a similar manner to how they inherit her dominance rank (baboons [40]; macaques [41]; vervet monkeys *Chlorocebus pygerythrus* [42]). For example, young infant rhesus macaques who do not move independently from their mother share her network [43], which can last up to seven months of age. When older, infants' social networks may remain linked to that of their mother even after they start to move independently. Such social preferences may be passed passively through social learning (i.e. observation of maternal social preferences) and/or familiarity (i.e. exposure to maternal social partners) [41], or through active maternal intervention (i.e. encouraging relationships with certain individuals) [44]. A modelling approach confirmed that a purely genetic explanation is not necessary [45].

Social bonds could also be affected by age. Immatures tend to spend less time with their mothers and socialize with same-sex age peers as they grow older, thus expanding and differentiating their social network from the maternal one [43,46]. This social exploration phase may lead to an increase in the number of partners immatures associate with [28,47], which may also derive from the greater tolerance that primates show towards immature individuals compared to adults [48,49]. Association with age peers (baboons [50]; macaques [51]; vervet monkeys [52]), same-sex individuals (baboons [49]; macaques [53]) or individuals who are matched by both age and sex (baboons [40]; blue monkeys *Cercopithecus mitis* [54]; diademed sifakas *Propithecus diadema* [55]) seems to be common to social primates when immature, although some exceptions occur (e.g. a decrease in association with peers [56]; or association with the opposite sex [55]).

Finally, the development of social connections could depend on sex. In general, immature females spend more time with the mother and maternal relatives during development than males [57,58] (but see [59]), both in female-philopatric species (baboons [49]; geladas *Thercopithecus gelada* [60]; macaques [61]; vervet monkeys [62]) and male-philopatric species (chimpanzees [63]; spider monkeys *A. geoffroyi* [64]). Daughters’ social connections may also resemble maternal ones more than sons' (macaques [53]). A species’ social system could drive sex differences in integration in the social network, in terms of both the number and strength of social bonds and the diversity of social partners [57,58]. In female-philopatric species, juvenile females typically dedicate more time to grooming and associate with more social partners than males (baboons [65]; macaques [66]; blue monkeys [54]; vervet monkeys [42]; but see [60,67]). Despite a general tendency to associate with same-sex peers with age, this becomes more marked in males than females. In fact, females tend to associate with a wider variety of partners, thus remaining more central in the group's network, while males tend to become more peripheral prior to dispersal by preferring male peers (baboons [68]; macaques [69]; blue monkeys [54]). Opposite tendencies were reported in male-philopatric species, in which grooming is equally common in males as in females and males have a wider social network than females (chimpanzees [70]; spider monkeys [64]).

Although most studies agree on how social networks change with age and sex, they largely focus on cross-sectional comparisons [54,69], rather than following the same cohort of individuals longitudinally (but see [42,66]). Compared to cross-sectional comparisons, longitudinal studies have the potential to identify developmental trends. In addition, many studies concern food-provisioned populations (e.g. at Cayo Santiago [66]), with a few exceptions targeting wild populations [42,54]. Wild populations may better represent the constraints faced by immature individuals in establishing social bonds. This is because primates trade grooming for tolerance during foraging, meaning that social bonds can be affected by competition for food [71,72], which may be different in food-provisioned populations compared to wild populations [73]. Finally, the transmission of maternal networks in female-philopatric species has been explored mainly in daughters [41,42] because of the stronger bond shared with the mother compared to sons [74]. However, males' integration in the maternal network is important to better understand possible constraints on information transmission across the group. For example, given that males tend to learn asocially, while females rely more on social learning [75], social information flow may be constrained if individuals who tend to produce information (males) have poor connections with the ones who tend to acquire it (females) [76]. For male primates, who are generally larger than females, it may be relatively more advantageous to explore, for example, a new, potentially higher pay-off, food patch or item because they can supplant a smaller individual, acquiring its resource, if unsuccessful [75]. Instead, females, who bear relatively higher parental investment costs [77], may be less willing to forego known foraging opportunities for an uncertain reward [19].

With this study, we aimed to contribute to the understanding of how animal social networks, and thus opportunities for social learning, change with age throughout the immature period, and how they differ between the sexes. We tested two main hypotheses about the development of social connections in wild immature baboons, using social network analysis in the context of a longitudinal study. Our first hypothesis (H1) was that immatures would ‘inherit' their mothers’ social networks at birth [45] and that these would differentiate during development [51]. We had three predictions for this hypothesis. P1a: as reported in other female-philopatric primate species [58], the time spent with the mother will decrease with age and this decrease will be greater for males than for females. P1b: while mother–offspring social networks will be positively correlated [8], the strength of the correlation will decrease with age as immatures become more independent from the mother [43]. The decrease will be greater for males than for females [61]. P1c: because the mother and maternal relatives have a special importance in matrilineal species [78,79], immatures' networks will be more correlated to the mothers’ networks than to those of other individuals [60]. This pattern will decrease with age and the decrease will be greater for males than for females [66].

Our second hypothesis (H2) was that an individual's social network characteristics would change during development in terms of both immatures' integration in the troop's network and the type of social partners with whom immatures associate [58]. We had three predictions for this hypothesis. P2a: the association with different social partners will increase with age as immatures widen their social network beyond the maternal network [47]. As observed in other female-philopatric primate species [58], this increase will be relatively lower in males, who will also have fewer social partners than females [42]. P2b: if immatures inherit their mothers' networks (P1b and P1c), their positions in the social network will remain stable throughout development for females, while males will become more peripheral with age [69]. P2c: the characteristics of immatures’ social partners will shift from a majority of adult partners (deriving from the maternal network) towards including more same-sex age peers or other immature individuals. This tendency will be greater for males than for females [54].

## 2. Methods

### 2.1. Study site and species

The study was carried out at the Tsaobis Baboon Project [80], a long-term (since 2000), individual-based study located on the edge of the Namib Desert, in Namibia (22°22′S, 15°44′E). The study area includes the large ephemeral Swakop River and its tributaries, with dense patches of riparian woodland surrounded by rocky hills, where vegetation is scarce and limited to dwarf trees and small shrubs (see [81] for a full description of the area).

Adult female chacma baboons give birth to an infant approx. every 2 years and infants are weaned around 12–18 months of age at Tsaobis [82]. For the purposes of this study, individuals less than or equal to 5 years old were considered immatures. We chose this threshold because, in most classifications, males are considered juveniles until 5 years old, entering sub-adulthood (before reaching sexual maturity) after 5 years old; females are considered juveniles until 4–5 years old and conceive their first infant around 6 years old [83–86]. Immature individuals less than or equal to 12 months old are referred to as infants, while those greater than 12 months old are considered juveniles [46].

### 2.2. Data collection

Observations were conducted on three troops (J, L and M) during six annual field seasons between 2014 and 2019. Data collection occurred over two- to three-month periods in each field season (approx. two months in 2014, 2016 and 2017; five months in 2015; three months in 2018 and 2019). Troops J and L were followed during all years, while M, which fissioned from J troop between 2016 and 2017, was followed only in 2017 and 2019. During the study periods, baboons were usually followed daily from dawn to dusk, and data were recorded on all recognizable individuals (see below) with the application CyberTracker [87].

The study population is habituated to the presence of observers on foot and the majority of the individuals were individually recognizable, apart from the unmarked juveniles and infants (the proportions of recognizable individuals were: 0.8 in 2014 and 2017; 0.7 in 2015; 1 in 2016 and 2019; and 0.9 in 2018). We quantified individuals' associations based on a proximity and an interaction (i.e. grooming) rule. Networks built with different rules may provide different information [88,89] and thus allow a more comprehensive description of baboons’ social network development. In addition, both spatial and grooming networks predicted social information use in this population [19].

Proximity associations were characterized through instantaneous (scan) sampling [90]. Because baboon troops at Tsaobis can spread over large distances with frequent mixing of individuals, it is difficult to perform surveys of the troop (the whole social group) at one point in time, as is done for some other species [91,92]. To guarantee sampling independence, we instead recorded the neighbours of ‘focal’ individuals, who were chosen from a randomized list including all the baboons present in the troop. All individuals within 10 m of a focal individual were recorded as a proximity ‘subgroup’. Individuals who did not have a neighbour within 10 m were recorded as alone. When a focal individual was sampled, s/he was not re-sampled within an hour if appearing again in a (new) list. Similarly, when an individual was recorded as a neighbour of a focal individual, s/he was not re-sampled as a focal individual within an hour. If group membership did not change substantially within an hour (during long resting periods, for example), observations ceased until group movement (and thus new subgroups) started again. Since very young infants are carried exclusively by their mothers, they were included in the scans only from when they were observed without the mother, indicating that they could move independently from her [19]. The number of individuals sampled in proximity scans varied across the study years, ranging from 44 to 59 for J troop, from 41 to 74 for L troop and remaining stable at 21 for M troop. In each month, each individual was sampled 4–13 times (averaged per year) in J troop (300–701 total observations per month—averaged per year), 8–18 times in L troop (423–1037 total per month) and 13–20 times in M troop (273–427 total per month).

Grooming interactions were recorded *ad libitum* [90] while moving through the troops. In this way, the *ad libitum*-collected data were not biased towards the individuals who were easier to find and, as such, we did not account for individual baboon observability. We collected data on the identities of the baboons grooming and the directionality of grooming events (giver to recipient). To avoid pseudo-replication, a grooming dyad was not recorded more than once within the same half-hour [19]. The number of grooming individuals varied across the study years, ranging from 36 to 62 for J troop, from 42 to 81 for L troop and from 21 to 24 for M troop. In each month, each individual groomed 5–34 times (averaged per year) in J troop (129–706 total grooming events per month—averaged per year), 12–52 times in L troop (444–1372 total per month) and 21–31 times in M troop (272–378 total per month).

Immature individuals' ages (in months) were determined by known and estimated dates of birth. Known dates were from births that occurred during fieldwork. If the birth occurred outside of the field season, the date of birth was estimated based on (i) possible conception dates from consortship records (i.e. mate-guarding events at the peak of a female's fertility) [93] after which the female did not menstruate, and assuming a pregnancy duration of six months [94]; or (ii) if the mother's conceptive cycle was not observed, through observation of the infant's coloration [95]. The identity of an infant's mother was determined through direct behavioural observations (nursing). The mothers' numbers of offspring in the troop during each season were calculated based on long-term troop composition data.

Dominance ranks were determined for adult females through dominance interactions recorded *ad libitum*. These included displacements, supplants, threats, chases and attacks for which a giver and recipient were identified. Only one interaction was recorded even if this involved several dominance behaviours in sequence. Female ordinal ranks were computed at the end of each season through the ‘I & SI’ method [96] using Matman 1.1.4 (Noldus Information Technology 2003). The method is appropriate for linear dominance hierarchies [96,97], and it has been applied to other studies on this population (e.g. [98]). The dominance hierarchies obtained for each season were strongly linear. We standardized absolute ranks by controlling for group size using the formula 1 − [(1 − *r*)/ (1 − *n*)], where *r* is an individual's absolute rank and *n* is the group size. Relative ranks range from 0 (lowest) to 1 (highest) [98].

### 2.3. Data analyses

Data analyses were performed with R v.4.0.2 [99]. Following previous studies with sparse network data [100–103], we used multiple static networks (i.e. describing a network sampled over a particular length of time) aggregated in approx. 30-day time periods. The periods chosen for time-aggregated networks should be appropriate for the kind of questions investigated and the available data resolution [20,104]. We chose these periods of time because immatures undergo rapid developmental changes during their first year of life [46]: this 30-day resolution allowed us to capture social network transformations during infancy as well as juvenile years.

Proximity and grooming data were treated separately. The sampling periods were generated starting from the first day in which data were collected for a given field season; these dates were different for proximity and grooming data, thus generating different windows and numbers of periods overall. In J troop, this resulted in 12 and 16 30-day periods for proximity and grooming data, respectively (1–4 periods per year depending on field seasons' lengths); in L troop, there were 11 and 17 30-day periods for proximity and grooming data, respectively (1–5 periods per year); in M troop, we produced two and three 30-day periods for proximity and grooming data, respectively (1–2 per year). To have a sufficient number of observations per individual to build robust and accurate networks, we removed any period with less than 95 observations. We also removed any individual who was seen less than five times per season (e.g. disappeared mid-season due to death or emigration). The removal of individuals for whom few data are collected is recommended because their network may not be represented accurately [104].

Social networks were produced for each sampling period, within each troop. We built weighted networks in which network values indicate both the presence and strength of a connection between two individuals [105], and, in the case of grooming, weighted and directed networks. We did not filter associations based on edge weight because thresholding (i.e. removing the associations that have a weight lower than the threshold) eliminates weak ties, which can nevertheless be important [106,107], and may generate errors in understanding the determinants of network structure [104]. We used a simple ratio index (SRI) to calculate weighted connections in the proximity network. SRI is the proportion of time two individuals associated (ranging between ‘0’—never associated—and ‘1’—always associated). This index is recommended only when individuals can be detected and identified correctly [108] as is the case at Tsaobis where group membership is stable, and visibility is usually excellent due to low vegetation cover. For the grooming network, we used grooming counts or proportions depending on the model tested (see below). Network graphs were computed from association networks and matrices of grooming counts using *igraph*'s *graph_adjacency* function. The grooming network was divided into its *out* (given) and *in* (received) components, which were treated both separately and united (total). Although proximity and grooming networks were analysed separately, similar models were applied in both cases, with the dependent variable differing depending on the network. All analyses were run on ego-networks: the variables tested were calculated for each immature individual in each sampling period [100,103].

To test our predictions, we used both linear mixed effects models (LMMs) and generalized linear mixed effects models (GLMMs) using the function *(g)lmer* from the package *lme4*. The package *lmerTest* was required to obtain *p*-values from models' summaries. The models are summarized in table 1. All models included the following fixed and random factors. The independent variables (fixed factors) of interest were age, sex and the interaction between age and sex. We then controlled as a fixed factor the mothers’ relative dominance rank because rank is known to affect social bonds in primates [39,109,110]. We also controlled as a fixed factor the mothers' number of offspring in the troop because the mothers’ grooming time might have to be shared among her offspring [111]. In addition, the number of maternal siblings may influence, for instance, immatures' grooming allocations in case they prefer maternal kin to other individuals [66,112]. In order to control for repeated measures from the same troops, we added troop as a fixed factor (because only three troops were followed in this study, it could not be included as a random factor [113]). Because our data have repeated measures from the same individuals through time, in each model, we included as a fixed factor the individuals’ identity (immature and mother) [101–103]. We nested immatures' identities within their mothers' identities because maternal behaviour could affect the offspring's sociality [114]. Finally, we added year as a random factor to consider between-years variation in environmental factors. We did not control for season in our analyses because most observations were made in the austral winter and baboons have no distinct breeding and mating seasons like, for example, macaques [86,106].

**Table 1.** Summary of the models used to test the predictions of hypotheses 1 (H1) and 2 (H2), including the dependent variable, the models’ fixed and random factors, and the type of test used. Under model, the asterisk (*) means that a permutation test was performed. Support indicates whether the prediction was supported and, if not or only partially, how.

hypothesis	prediction	model	dependent variable	network	support	test	fixed/random factors
H1: immatures would inherit their mothers' social networks at birth, and these would differentiate throughout development	P1a: time spent with the mother will decrease with age and this decrease will be greater for males than for females	MP1a	proportion of time with mother	proximity	yes	GLMM binomial	fixed: age, sex, age:sex, nb. offspring, relative rank, troop random: year, immatures’ identities nested within their mothers' identities
				grooming	total: yes		
	P1b: while mother–offspring networks will be positively correlated, the strength of the correlation will decrease with age. This decrease will be greater for males than for females	MP1b*	mother–offspring network correlation	proximity	yes	LMM	
				grooming	given: partial (↑ with age in females)		
					received: partial (age:sex not significant)		
					total: yes		
	P1c: immatures' networks will be more correlated to the mothers' networks than to those of other individuals. This pattern will decrease with age and the decrease will be greater for males than for females	MP1c.1*	0–1 score: 1 = correlation with mother's network > correlation with random individual's network; 0 = vice versa	proximity	no (age and age:sex not significant)	GLMM binomial	
				grooming	given: no (↑ with age, age:sex not significant)		
					received: no (age and age:sex not significant)		
					total: no (age and age:sex not significant)		
		MP1c.2*	0–1 score: 1 = correlation with mother's network > mean correlation with other individuals; 0 = vice versa	proximity	no (↑ with age, age:sex not significant)		
				grooming	given: no (↑ with age, age:sex not significant)		
					received: no (age and age:sex not significant)		
					total: no (↑ with age, age:sex not significant)		
H2: social network characteristics would change throughout development, in terms of both the position in the network and the type of social partners with whom immatures associate	P2a: the associations with different social partners will increase with age as immatures widen their social network. This increase will be relatively lower in males, who will also have fewer social partners than females	MP2a*	strength	proximity	no (↓ with age, sex and age:sex not significant)	LMM	fixed: age, sex, age:sex, nb. offspring, relative rank, troop, strength mother random: year, immatures' identities nested within their mothers' identities
				grooming	given: yes		
					received: partial (sex and age:sex not significant)		
					total: partial (sex not significant)		
	P2b: immatures' positions in the social network will remain stable throughout development for females, while males will become more peripheral with age	MP2b.1*	eigenvector centrality	proximity	no (↓ with age for females and age:sex not significant)	LMM	fixed: age, sex, age:sex, nb. offspring, relative rank, troop, centrality mother random: year, immatures' identities nested within their mothers' identities
				grooming	total: no (↑ with age for both sexes and age:sex not significant)		
		MP2b.2*	betweenness centrality	proximity	no (age and age:sex not significant)	LMM	fixed: age, sex, age:sex, nb. offspring, relative rank, troop, betweenness mother random: year, immatures' identities nested within their mothers' identities
				grooming	total: no (↑ with age for both sexes)		
	P2c: the characteristics of immatures' social partners will shift from a majority of adult partners to a majority of same-sex age peers or other immature individuals. This tendency will be greater for males than for females	MP2c.1*	proportion of immature (less than or equal to 5 years old) partners	proximity	partial (age not significant)	GLMM binomial	fixed: age, sex, age:sex, nb. offspring, relative rank, troop random: year, immatures' identities nested within their mothers' identities
				grooming	given: partial (age:sex not significant)		
					received: partial (age:sex not significant)		
					total: partial (age:sex not significant)		
		MP2c.2*	proportion of peer (± 6 months) partners	proximity	no (age and age:sex not significant)		
				grooming	given: partial (age:sex not significant)		
					received: partial (age:sex not significant)		
					total: partial (age:sex not significant)		
		MP2c.3*	proportion of same-sex partners	proximity	yes		
				grooming	given: partial (age:sex not significant)		
					received: yes		
					total: yes		

The models (described in further detail below) were presented as ‘full models’: the models did not undergo any simplification procedure after the *a priori* variable selection based on the current literature (see above and table 1) [115,116]. We obtained *R*^2^ values (coefficient of determination) from the package *MuMIn* and the function *r.squaredGLMM*. We checked for multi-collinearity using variance inflation factors (VIFs) with the package *car* and the function *vif*. For LMMs, we checked residuals' homoscedasticity. For GLMMs, we verified overdispersion with the package *DHARMa* and the function *testDispersion*. In all cases, VIF values were less than or equal to 3, indicating low collinearity [117,118]; LMM models’ residuals were normally distributed and GLMMs' under/overdispersion, when significant, was within the range of 0.7–2, indicating a normal degree of variation for a binomial distribution [119]. For binomial models, we obtained the (i) odds ratios (OR) by exponentiating the variables’ estimates and (ii) 97.5% confidence intervals through the function *confint.merMod* (*method* = *‘Wald’*). We then calculated the percentage variation in odds per each unit variation in a dependent variable as (*OR* − 1) × 100 [120]. We expressed differences in OR changes from one age stage (*t*_0_) to the next (*t*_1_) as increase/decrease percentages, computed through the formula: [(*ORt*_1_ − *ORt*_0_)/*ORt*_0_)] × 100. For LMMs, estimates (beta coefficients) were described as increase/decrease percentages over a dependent variable's range. In the presence of an interaction, the main effects were interpreted as conditional effects [121,122]. To compute age changes in effect sizes, we chose two specific periods based on the literature: (i) 1 year old, when immatures are generally weaned but still spend most of the time with the mother [46]; (ii) 4 years old, when immatures should have established their own social relationships, mostly independently from maternal presence [66]. To obtain differences in effect sizes between sexes, we calculated the effect of sex at 30 months of age (the middle of the immatures' age range). Finally, to compare the effects of different independent variables (within a model), we derived standardized (i.e. all the variables are on the same scale) estimates (see electronic supplementary material, §6). In all cases, *p*-values ≤ 0.05 were considered significant [115,123].

Social network data are non-independent because the data are relational. Data permutations serve to solve the issue of data non-independence when performing hypothesis testing with network data [104,105]. The permuted data are used to re-run the model to obtain a ‘null’ effect size against which the observed effect size can be compared. For the proximity data, in most cases, we permuted the datastream using the *network_swap* function from the *asnipe* package; however, node-based permutations (see below) were used for three of the models (as described further on). Datastream permutations randomize subgroup membership by swapping observations between individuals in the N × K matrices (i.e. N individuals per K observations) [124] and are recommended for group membership data [123]. The original datastream was used to make the first permutation, swapping 20 observations in each 30-day period. For each successive permutation, the previous permuted matrix was re-permuted. In this way, matrices become more and more randomized compared to the original datastream. For this reason, a set of initial permutations was discarded. We made 300 permutations per period, discarded the first 200 permuted matrices, retaining 100 randomized networks/period to be used in the ‘randomized’ models. These permuted matrices represent the null hypothesis of random associations among individuals [123]. For the grooming data and three models from proximity data (P2c models; see details below), we applied node-based permutations, which are commonly used for grooming networks [28,118], with the function *rmperm* from the package *sna*. Node-based permutations re-distribute the nodes (individuals) while leaving the same number of associations [104]. As for the proximity data, we made 300 permutations per sampling period and selected the last 100 for each period. To determine the statistical significance of the estimated social network effect, we re-ran each model (see below) using the 100 permuted matrices. For each model and each network variable, the two-tailed *p*-value was calculated as the number of times that the observed effect size was larger or smaller than the distribution of effect sizes from the permuted data (resulting as either *p*-value = 0.05 or not significant) [123].

### 2.4. H1. Inheritance of maternal social networks

From the base model, we tested our hypotheses and predictions using the following specific models (table 1). For H1, we ran a total of four sets (applied to the different networks) of models across our three predictions. First, we quantified changes in the time immatures spend with the mother (P1a), using binomial GLMMs (*family = binomial, link = logit*) [125] in which the dependent variable was the proportion of time spent with the mother (MP1a). For proximity, we used the proportion of observations in which each immature was observed with the mother (within 10 m) in each observation period; for grooming, we used the proportion of observations in which each focal immature groomed with the mother, considering only the total grooming. Permutations were not run for these models because the dependent variable (i.e. proportion of observations with the mother) is not a social network value or metric.

We next tested whether there was a positive correlation between mothers' and immatures’ social networks and how this changed through time (P1b). To this end, we calculated two measures of similarity between the mothers' and immatures’ ego-networks for each period (i.e. the vectors that correspond to the square matrix rows of each immature focal individual and her/his mother). The two measures were (i) the test statistic from a Pearson's correlation (bounded between −1 and 1) of the mother's and offspring's vectors [8] and (ii) the cosine similarity between the mother's and immature's vectors [42]. Because these two network similarity measures were positively correlated and the results of the models using either one of these dependent variables were qualitatively similar, we report here only the results for models using the Pearson's correlation (MP1b) (see electronic supplementary material, §1, for results using the cosine similarity). For this analysis, we used the proportion matrix for grooming because we were interested in similarities between networks, and proportions better represent the distribution of grooming to/from different partners, independent of the total number of events. Immatures' grooming networks may be constrained by partner availability if they tend to be in proximity to the mother and could thus choose only among her social partners. Controlling for the time spent in the mother's proximity would potentially solve this issue [126]. However, there was no significant effect of the proportion of time spent in proximity with the mother (see electronic supplementary material, §2). Because including this variable reduced our dataset (since proximity data were not available for every grooming period), it was not included in the final models.

We next tested whether immatures' ego-networks were more similar to their mothers’ ego-networks than to those of other group-mates and how this changed with age (P1c). Because there is no agreed method to test this, we adopted three different approaches, all of which compared the Pearson's correlation coefficient (*r*) of the mother–immature dyad with combinations of other dyads from the proximity networks or grooming proportions networks. We present two of these approaches here and discuss the third one in electronic supplementary material, §5. In the first approach, for each immature individual, we selected a random non-mother individual from the troop and created a new variable, assigning a score of ‘1’ when the mother–immature's *r* was higher than the random–immature's *r*, and ‘0’ when the reverse was true. We ran a binomial GLMM with the 1/0 score as the dependent variable (MP1c.1). In the second approach, we applied the same 1/0 scoring method and a binomial GLMM, using the mean *r* for all the non-mother individuals instead of a random individual (MP1c.2).

### 2.5. H2. Changes in social network characteristics throughout development

For H2, we ran a further six sets of models. Predictions P2a and P2b predicted changes in individuals' node-level social network metrics that reflect the level of integration in the troop's network. We chose to calculate individuals': strength (MP2a), eigenvector centrality (MP2b.1) and betweenness centrality (MP2b.2). Strength is the sum of the edge weights connected to a node, representing the relative association rate within a sampling period and thus an individual's opportunities to collect social information. Eigenvector centrality (hereafter centrality) is the sum of the centralities of an individual's social partners. An individual can have high centrality either by having a large number of partners or associating with individuals who have numerous connections (or both) [104,127]. This metric also describes an individual's relative opportunities to acquire and propagate social information in a network [19]. Betweenness centrality (hereafter betweenness) is the number of shortest paths that cross a node. This measures an individual's importance in connecting otherwise unconnected individuals [104]. For both proximity SRI and grooming count data, we calculated the metrics using *igraph* (functions *strength*, *eigen_centrality*, *betweenness*). Grooming given, received and total were computed for strength, while given/received components cannot be separated for centrality and betweenness. We analysed these five metrics using LMMs. Proximity betweenness and grooming strength given were log-transformed (*log1p* function) to make them normally distributed; grooming centrality and betweenness were square root transformed (*sqrt* function). In these models, we additionally controlled as a fixed factor the corresponding metric of the mother. This is because, if immatures inherit their mothers' networks, the mother's social network position may affect that of her offspring [42]. To make metrics comparable across networks of different sizes, metrics were standardized within each sampling period by mean-centring them around zero with a standard deviation of one through the function *scale* [128].

To test P2c, that immatures will shift from associating with adults to peers as they age, we counted the number of immature, peer and same-sex partners each immature associated with. An individual's number of social partners is their degree [104]. A peer was defined as an individual whose age was ±6 months of a focal individual's age. We distinguished between immatures and peers to understand whether immatures were generally attracted by other non-adult individuals (less than or equal to 5 years old) or if they specifically targeted partners of their same age. To test the tendency to associate with different partner categories with age, we ran binomial GLMMs with the proportion of immature (MP2c.1), peer (MP2c.2) and same-sex (MP2c.3) partners as dependent variables. Using the proportion of partners belonging to a certain category over the total number of partners of that category in the troop, we could control for partner availability [66]. For the P2c models, we ran node-based permutations for the proximity network. These were chosen over datastream permutations because, as mentioned, they keep the number of associations constant [104] and were thus appropriate to test whether there was a shift in partner preferences based on their phenotype rather than number.

## 3. Results

We collected a total of 12 592 proximity records (J: 5862; L: 6030; M: 700) and 19 597 grooming records (J: 6277; L: 12 398; M: 922). The number of immature individuals followed (i.e. sample size) were 109 to 116 for the proximity network and 93 to 142 for the grooming network, born from 32 to 43 mothers (see electronic supplementary material, §3, for the number of observations and the sample size of each model) [129].

**Figure 1.**
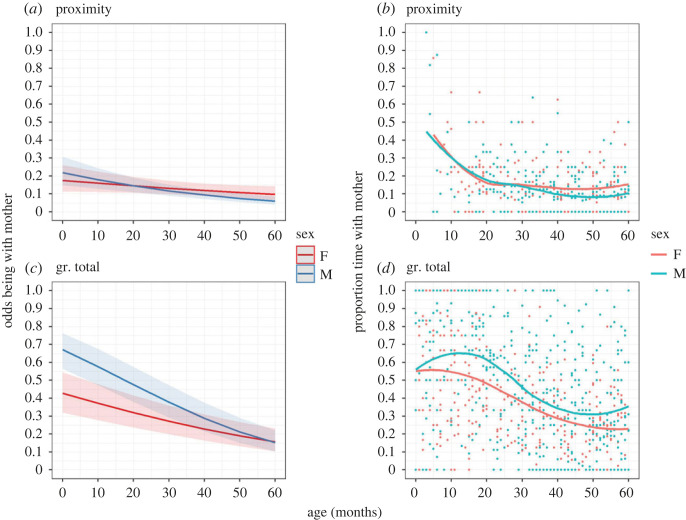
Changes in the immatures' (*a*,*c*) odds of being with the mother (model-predicted values, MP1a) and (*b*,*d*) proportion of time spent with the mother (raw data) with age in the two sexes (F = female, M = male) for the proximity and grooming (gr.) networks. Higher OR means a relatively higher likelihood of an event (*y*-axis variable) to occur. Ribbons represent 97.5% confidence intervals.

**Figure 2.**
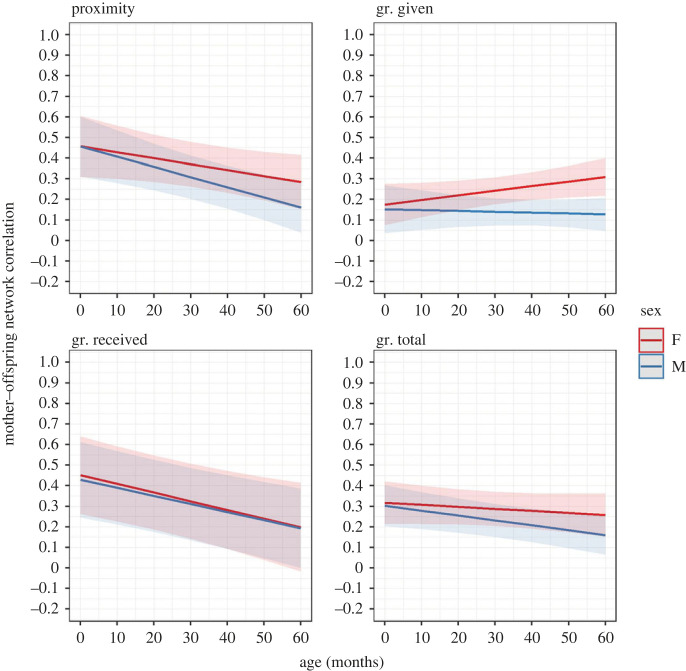
Changes in the mother–offspring social network correlation with age in the two sexes (F = female, M = male) for the proximity and grooming (gr.) networks (MP1b). Ribbons represent 97.5% confidence intervals.

**Figure 3.**
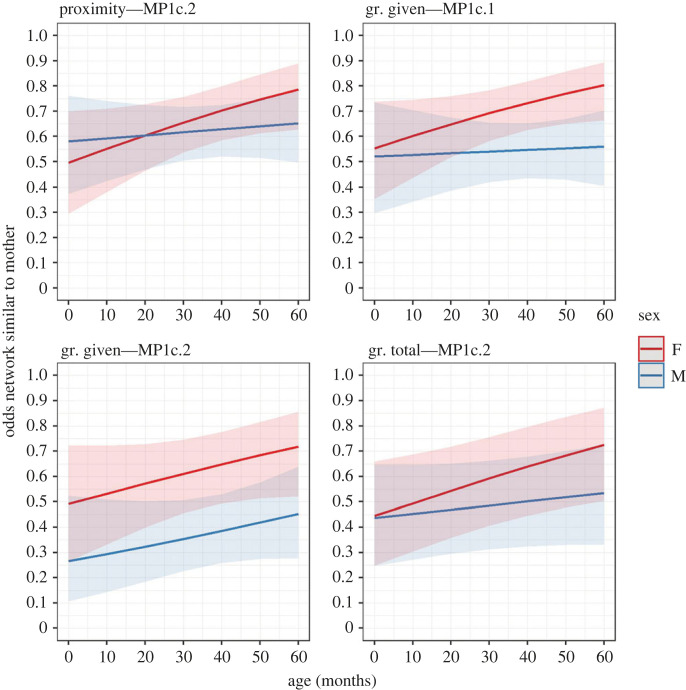
Changes in the odds that the correlation with the mother's network was higher than the correlation with a random individual's network (MP1c.1) and the mean correlation with other individuals' networks (MP1c.2). These changes are related to age and divided between the two sexes (F = female, M = male) for the proximity and grooming (gr.) networks. Higher OR means a relatively higher likelihood of an event (*y*-axis variable) to occur. Ribbons represent 97.5% confidence intervals.

**Figure 4.**
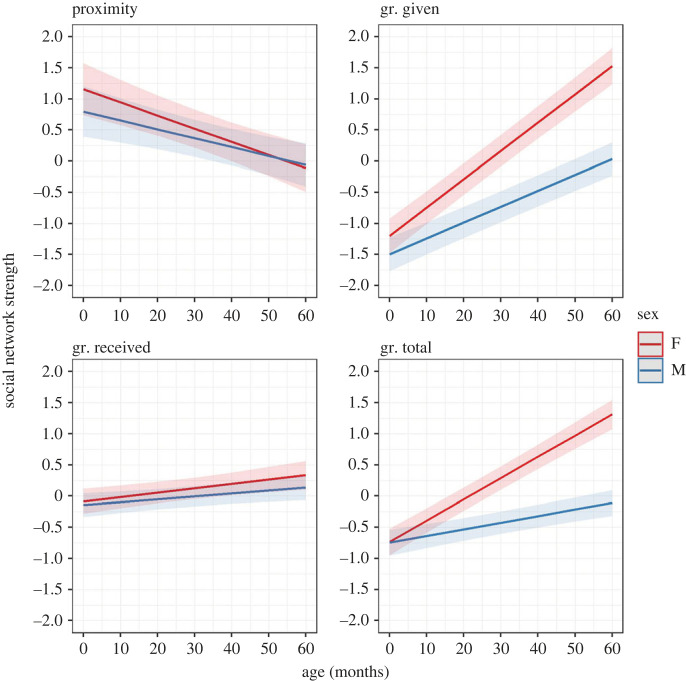
Changes in immatures’ social network strength with age in the two sexes (F = female, M = male) for the proximity and grooming (gr.) networks (MP2a). Ribbons represent 97.5% confidence intervals.

**Figure 5.**
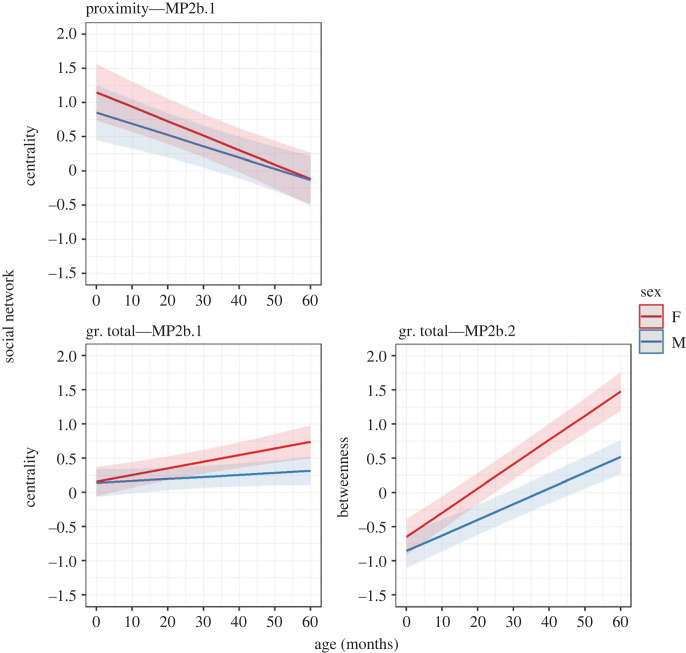
Changes in immatures' social network centrality (MP2b.1) and betweenness (MP2b.2) with age in the two sexes (F = female, M = male) for the proximity and grooming (gr.) networks. Ribbons represent 97.5% confidence intervals.

**Figure 6.**
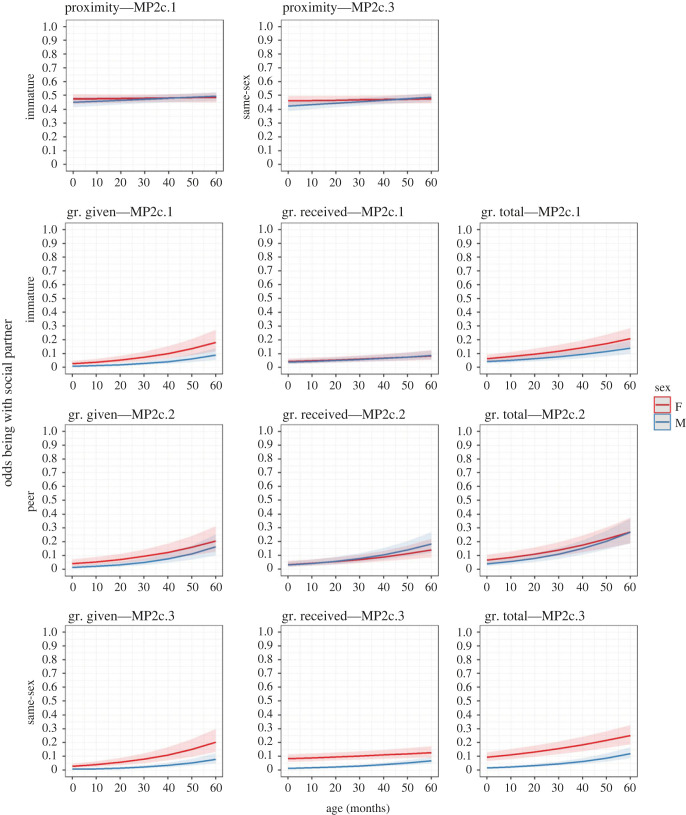
Changes in immatures’ odds of being with a particular social partner (another immature, MP2c.1; a peer, MP2c.2; a same-sex individual, MP2c.3) with age in the two sexes (F = female, M = male) for the proximity and grooming (gr.) networks. Higher OR means a relatively higher likelihood of an event (*y*-axis variable) to occur. Ribbons represent 97.5% confidence intervals.

In the following sections, we present the significant results (following each prediction) on our main variables of interest: age, sex and their interaction (tables 2 and 3; figures 1–6). Full tables are reported in the electronic supplementary material, §5 (together with the results of the third approach used to test P1c; see Methods). Comparisons among models' variables through standardized estimates are shown in electronic supplementary material, §6.

**Table 2.** Models' results on the age and sex (and their interaction) variables for the hypothesis 1 models (tables with all the independent variables are available in electronic supplementary material, §5). Under variable, age:sex indicates the presence of an interaction. The values reported for sex refer to males. The estimate, standard error (s.e.), *p*-value and conditional (delta) *R*^2^ are reported for each variable/model. The names of models that have significant variables and the rows of those significant variables are marked in italics.

model	variable	estimate	s.e.	*p*-value	*R*²
*P1a proximity*	*age*	*−0.0112*	*0.0051*	*< 0.05*	*0.27*
	sex	0.2702	0.2529	NS	
	*age:sex*	*−0.0135*	*0.0067*	< 0.05	
*P1a grooming total*	*age*	*−0.0232*	*0.0035*	*< 0.001*	*0.84*
	*sex*	*1.0043*	*0.2027*	*< 0.001*	
	*age:sex*	*−0.0173*	*0.0038*	*< 0.001*	
*P1b proximity*	*age*	*−0.0029*	*0.0013*	*0.05*	*0.20*
	sex	−0.0007	0.0670	NS	
	*age:sex*	*−0.0021*	*0.0018*	*0.05*	
*P1b grooming given*	*age*	*0.0022*	*0.0010*	*0.05*	*0.25*
	sex	−0.0227	0.0607	NS	
	*age:sex*	*−0.0027*	*0.0015*	*0.05*	
*P1b grooming received*	*age*	*−0.0042*	*0.0013*	*0.05*	*0.44*
	sex	−0.0222	0.0575	NS	
	age:sex	0.0003	0.0016	NS	
*P1b grooming total*	*age*	*−0.0010*	*0.0010*	*0.05*	*0.28*
	sex	−0.0147	0.0449	NS	
	*age:sex*	*−0.0014*	*0.0012*	*0.05*	
P1c.1 proximity	age	0.0053	0.0090	NS	0.03
	sex	−0.3070	0.4550	NS	
	age:sex	0.0017	0.0120	NS	
*P1c.1 grooming given*	*age*	*0.0198*	*0.0091*	*0.05*	*0.08*
	sex	−0.1326	0.4849	NS	
	age:sex	−0.0172	0.0126	NS	
P1c.1 grooming received	age	−0.0088	0.0092	NS	0.13
	sex	−0.4860	0.3657	NS	
	age:sex	0.0108	0.0115	NS	
P1c.1 grooming total	age	0.0096	0.0072	NS	0.17
	sex	−0.3061	0.3116	NS	
	age:sex	−0.0022	0.0092	NS	
*P1c.2 proximity*	*age*	*0.0219*	*0.0097*	*0.05*	*0.08*
	sex	0.3355	0.4720	NS	
	age:sex	−0.0168	0.0128	NS	
*P1c.2 grooming given*	*age*	*0.0161*	*0.0100*	*0.05*	*0.19*
	*sex*	*−0.9799*	*0.5681*	*0.05*	
	age:sex	−0.0024	0.0143	NS	
P1c.2 grooming received	age	−0.0112	0.0114	NS	0.25
	sex	−0.1466	0.4497	NS	
	age:sex	0.0021	0.0141	NS	
*P1c.2 grooming total*	*age*	*0.0198*	*0.0079*	*0.05*	*0.24*
	sex	−0.0312	0.3522	NS	
	age:sex	−0.0134	0.0101	NS

**Table 3.** Models' results on the age and sex (and their interaction) variables for the hypothesis 2 models (tables with all the independent variables are available in electronic supplementary material, §5). Under variable, age:sex indicates the presence of an interaction. The values reported for sex refer to males. The estimate, standard error (s.e.), *p*-value and conditional (delta) *R*^2^ are reported for each variable/model. The names of models that have significant variables and the rows of those significant variables are marked in italics.

model	variable	estimate	s.e.	*p*-value	*R*²
*P2a proximity*	*age*	*−0.0211*	*0.0039*	*0.05*	*0.41*
	sex	−0.3603	0.1936	NS	
	age:sex	0.0069	0.0050	NS	
*P2a grooming given*	*age*	*0.0455*	*0.0019*	*0.05*	*0.72*
	*sex*	*−0.2985*	*0.0893*	*0.05*	
	*age:sex*	*−0.0199*	*0.0025*	*0.05*	
*P2a grooming received*	*age*	*0.0069*	*0.0020*	*0.05*	*0.40*
	sex	−0.0613	0.0922	NS	
	age:sex	−0.0022	0.0025	NS	
*P2a grooming total*	*age*	*0.0341*	*0.0019*	*0.05*	*0.64*
	sex	−0.0125	0.0863	NS	
	*age:sex*	*−0.0235*	*0.0023*	*0.05*	
*P2b.1 proximity*	*age*	*−0.0211*	*0.0036*	*0.05*	*0.49*
	sex	−0.2964	0.1831	NS	
	age:sex	0.0047	0.0047	NS	
*P2b.1 grooming total*	*age*	*0.0097*	*0.0022*	*0.05*	*0.49*
	sex	−0.0195	0.1005	NS	
	age:sex	−0.0068	0.0028	NS	
P2b.2 proximity	age	0.0054	0.0042	NS	0.07
	sex	0.1166	0.2167	NS	
	age:sex	−0.0055	0.0057	NS	
*P2b.2 grooming total*	*age*	*0.0355*	*0.0023*	*0.05*	*0.52*
	sex	−0.2042	0.1020	NS	
	*age:sex*	*−0.0126*	*0.0029*	*0.05*	
*P2c.1 proximity*	age	0.0007	0.0013	NS	*0.14*
	*sex*	*−0.0922*	*0.0641*	*0.05*	
	*age:sex*	*0.0021*	*0.0017*	*0.05*	
*P2c.1 grooming given*	*age*	*0.0346*	*0.0032*	*0.05*	*0.64*
	*sex*	*−1.1815*	*0.1887*	*0.05*	
	age:sex	0.0060	0.0044	NS	
*P2c.1 grooming received*	*age*	*0.0119*	*0.0027*	*0.05*	*0.36*
	sex	−0.1137	0.1339	NS	
	age:sex	0.0023	0.0036	NS	
*P2c.1 grooming total*	*age*	*0.0227*	*0.0022*	*0.05*	*0.56*
	*sex*	*−0.4327*	*0.1195*	*0.05*	
	age:sex	−0.0010	0.0030	NS	
P2c.2 proximity	age	−0.0001	0.0026	NS	0.01
	sex	−0.0362	0.1412	NS	
	age:sex	0.0018	0.0036	NS	
*P2c.2 grooming given*	*age*	*0.0303*	*0.0051*	*0.05*	*0.31*
	*sex*	*−1.1021*	*0.3042*	*0.05*	
	age:sex	0.0137	0.0073	NS	
*P2c.2 grooming received*	*age*	*0.0261*	*0.0054*	*0.05*	*0.23*
	sex	−0.0887	0.2828	NS	
	age:sex	0.0067	0.0070	NS	
*P2c.2 grooming total*	*age*	*0.0275*	*0.0042*	*0.05*	*0.33*
	*sex*	*−0.5450*	*0.2297*	*0.05*	
	age:sex	0.0090	0.0057	NS	
*P2c.3 proximity*	*age*	*0.0010*	*0.0012*	*0.05*	*0.18*
	*sex*	*−0.1528*	*0.0627*	*0.05*	
	*age:sex*	*0.0032*	*0.0016*	*0.05*	
*P2c.3 grooming given*	*age*	*0.0364*	*0.0029*	*0.05*	*0.70*
	*sex*	*−1.5735*	*0.1916*	*0.05*	
	age:sex	0.0078	0.0044	NS	
*P2c.3 grooming received*	*age*	*0.0080*	*0.0023*	*0.05*	*0.57*
	*sex*	*−1.9513*	*0.1537*	*0.05*	
	*age:sex*	*0.0210*	*0.0038*	*0.05*	
*P2c.3 grooming total*	*age*	*0.0197*	*0.0020*	*0.05*	*0.72*
	*sex*	*−1.8453*	*0.1327*	*0.05*	
	*age:sex*	*0.0158*	*0.0032*	*0.05*	

### 3.1. H1. Inheritance of maternal social networks

To address our first hypothesis, we tested how the time spent with the mother (P1a) and the correlation between the offspring's and mothers’ social networks (P1b and P1c) changed through time. Our first prediction for H1, P1a, was confirmed both for the proximity and grooming total networks (table 2 and figure 1): immatures spent significantly less time with their mothers as they grew older, and this decrease was significantly greater in males than in females. Between 1 and 4 years old, the odds of being in proximity with the mother decreased by 33.27% and 58.89% in females and males, respectively; the odds of grooming (total) with the mother decreased by 56.63% and 76.74% in females and males, respectively. However, males groomed significantly more with their mothers than females (not predicted): at 30 months old, the odds of grooming (total) with the mother were 62.46% higher in males compared to females.

Our second prediction for H1, P1b, was partially confirmed, depending on the network (table 2 and figure 2). The mean correlation values between the mothers' and offspring's networks were positive for both the proximity and grooming networks (see electronic supplementary material, §4). As predicted for the proximity network, the immatures' networks became significantly less similar to their mothers’ with age and this decrease was significantly greater in males compared to females. Between 1 and 4 years old, the mother–offspring proximity network correlation decreased by 7.2% and 12.6% in females and males, respectively. For the grooming network, the support to P1b was less clear compared to the proximity network. According to our prediction for grooming received and total (as for the proximity network), the immatures' networks became significantly less similar to their mothers’ with age. This pattern was significantly more marked in males than in females only for grooming total. Between 1 and 4 years old, the mother–offspring grooming network correlation decreased by 12.24% and 2.88% (received and total, respectively) in females, and by 11.52% and 6.84% (received and total, respectively) in males. Instead, contrary to our prediction for females, age had significant opposite effects in the two sexes for grooming given: between 1 and 4 years old, the mother–offspring network correlation increased by 6.84% in females and decreased by 1.08% in males.

Our third prediction for H1, P1c, had contrasting support depending on the network and the model (table 2 and figure 3). The observations in which the immatures' proximity and grooming networks were more similar to those of their mothers (compared to the networks of other individuals) were higher than the observations in which the opposite was true (less similar to their mothers’ networks) in nine (of 16) models (see electronic supplementary material, §4). Contrary to our prediction for the proximity network, we found evidence that immatures' networks became significantly more similar to their mothers’ compared to those of other individuals with age in MP1c.2. This pattern was less marked in males than in females, but the age/sex interaction was not significant. Between 1 and 4 years old, the odds of having a proximity network relatively more similar to the mother's one increased by 119.79% and 20.07% in females and males, respectively. Age and the age/sex interaction were not significant in MP1c.1. For the grooming network, the models' support on age changes was less clear compared to the proximity network. Among the six models tested for the grooming network, age and the age/sex interaction were significant in three models (not significant in MP1c.1 and MP1c.2 received, and in MP1c.1 total). In contrast with our prediction (as for the proximity network), the immatures’ grooming given (MP1c.1 and MP1c.2) and total (MP1c.2) networks became significantly more similar to their mothers' compared to other individuals. This pattern was less marked in males, although the age/sex interaction was not significant. Between 1 and 4 years old, the odds of having a network relatively more similar to the mother's one increased between (hereafter—range of values) 78.44 and 104.32% (MP1c.2 given and MP1c.2 total, respectively) in females and between 9.89 and 63.67% (MP1c.1 and MP1c.2 given, respectively) in males. Finally, in MP1c.2, males' grooming given networks matched those of their mothers significantly less than the ones of other individuals compared to females (not predicted): at 30 months of age, the odds of having a network relatively more similar to the mother's one were 65.07% lower in males than in females.

### 3.2. H2. Changes in social network characteristics throughout development

To address our second hypothesis, we explored how immatures' integration in the troop's network changed during development (relative association rates with different social partners—P2a—and overall network position—P2b) and modelled shifts in social partner preferences (P2c). Our first and second predictions for H2, P2a and P2b, were partially confirmed, depending on the network and the metric involved (table 3; figures 4 and 5). Contrary to what we predicted for the proximity network, both strength (MP2a) and centrality (MP2b.1) significantly decreased as immatures grew older and this pattern was more marked in females, although the age/sex interaction was not significant. Between 1 and 4 years old, proximity strength and centrality decreased, respectively, by 14.4% and 12.96% in females, and by 9.72% and 10.08% in males. Finally, proximity strength was not significantly predicted by sex, and betweenness (MP2b.2) was not significantly predicted by age and the age/sex interaction. Instead, for the grooming network, immatures' strength (all components, as predicted), centrality and betweenness (total, contrary to our prediction) significantly increased with age. According to our prediction, the increases in grooming strength (all components), centrality and betweenness (total) were less marked in males. However, the age/sex interaction was not significant for strength received and centrality total. Between 1 and 4 years old, grooming strength increased by 37.44% and 5.76% (given and received, respectively) in females, and by 20.88% and 3.96% (given and received, respectively) in males. At the same time, grooming centrality and betweenness (total) increased, respectively, by 5.76% and 30.24% in females, and by 1.8% and 19.44% in males. Finally, as predicted, males had a lower grooming given strength than females, while sex was not significant for grooming received and total: at 30 months old, males’ strength given was 6.83% lower than females'.

Our third prediction for H2, P2c, was partially confirmed, depending on the network (table 3 and figure 6). As predicted for the proximity network, immatures shifted their social preferences towards immature (MP2c.1) and same-sex (MP2c.3) partners while growing older. This age change was significant only for MP2c.3, while the pattern was significantly more marked in males than in females in both models. Between 1 and 4 years old, the odds of being in proximity with an immature and a same-sex partner increased, respectively, by 2.43% and 3.51% in females, and by 10.57% and 16.28% in males. Instead, the change in preference for peer partners (MP2c.2) with age was not significant for the proximity network. As predicted for the grooming network, immatures developed a significant preference for grooming immature and peer partners with age (all components). This pattern was relatively more marked in males, but the age/sex interaction was not significant. Between 1 and 4 years old, the odds of grooming with an immature and a peer partner increased between (hereafter—range of values) 53.57 and 247.3% (MP2c.1 received and given, respectively) in females and between 66.93 and 387.93% in males (MP2c.1 received and MP2c.2 given, respectively). Moreover, as predicted, immatures groomed significantly more with partners of the same sex as they aged (all components). This pattern was more marked in males than in females, although the age/sex interaction was significant only for grooming received and total. Between 1 and 4 years old, the odds of grooming with a partner of the same sex increased between 33.52 and 270.69% (received and given, respectively) in females and between 184.75 and 391.22% (received and given, respectively) in males. Finally, sex had a significant effect in all MP2c models but in MP2c.2 proximity, and in MP2c.1 and MP2c.2 grooming received (not predicted). Males were less in proximity and groomed less with partners of similar age and of the same sex than females. At 30 months of age, the odds of being in proximity with an immature and a same-sex partner were, respectively, 2.80% and 5.43% lower in males than in females; the odds of grooming with an immature, a peer or a same-sex partner were lower in males than in females between 24.04 and 74.64% (MP2c.2 and MP2c.3 total, respectively).

## 4. Discussion

In this paper, we explored how wild chacma baboon social networks are shaped throughout the immature period, focusing on age changes and sex differences. Here, we first discuss the effects of age and sex (and their interaction) on the inheritance of maternal networks (H1) and on the changes in the immatures’ social network integration and social partners' characteristics (H2). We then comment on the potential implications of these developmental changes and sex differences in network patterns for social information transmission.

Immature baboons inherited their mothers’ social connections (H1). As they aged, they reduced the strength of their relationship with the mother and maternal social partners, but a mother's bonds continued to influence her offspring's throughout their development. The gradual acquisition of ecological competence (e.g. knowing what to eat) may decrease immatures' reliance on maternal care [16,130] and consequently the time spent with the mother, as is common among post-weaning mammals [131,132]. We found that this was the case, as immature baboons spent less time with their mothers with age. Perhaps as a result, immatures’ networks tended to diverge from those of their mothers with age. Nevertheless, mother–offspring proximity and grooming networks were positively correlated and immatures' networks generally became more similar to their mothers’ compared to those of other individuals with age, thus supporting a social inheritance explanation [8,41]. Although the mean mother–offspring network correlations were relatively low (less than 0.5; see electronic supplementary material, §4), these values may be normal given that (i) immatures' grooming integration increased with age (see Results §H2), being thus initially lower than that of their mothers, and (ii) in wild populations social networks are affected by demographic changes [133,134]. Our mean cosine similarity values for immature female grooming networks were qualitatively similar to those reported for vervet monkeys [42] (see electronic supplementary material, §1). The relatively low cosine similarity values in vervet monkeys were hypothesized to derive from the changes in the mothers’ own grooming networks, which constituted a moving target for their daughters [42]. The same process may contribute to explain our results, given that the mothers' grooming networks changed between the study years (see electronic supplementary material, §7).

Although immature baboons inherited their mothers’ social networks, our results showed that their social network integration and social partner preferences changed throughout development (H2). Age was an important determinant of this process (see electronic supplementary material, §6). Given that the mother's presence constrains the number and kind of partners with whom immatures can associate [135,136], age, which determines the extent of an immature's dependence on the mother [46], could be an important predictor of these network changes. In particular, as they aged, immatures had lower associations in their proximity network but were more active in grooming. First, the decrease in proximity network integration may be due to both external factors and immatures' partner choices. External factors could include primates’ attraction towards infants [46,51] and the greater tolerance that adults have towards younger versus older immatures. The infant network may shrink as older infants lose their attractiveness [137,138]. In addition, younger immature primates are generally granted relatively higher proximity tolerance [15], which could aid their survival through the acquisition of ecological knowledge [48,139] and protection [39,114]. On the other hand, older immatures may need relatively less support and be willing to associate with fewer partners [49,140]. Alternatively (or concurrently), immatures' proximity integration may decrease because they become more ‘socially selective’. Social selectivity has been reported in mammals during senescence and may result from several factors, such as reduced energetic and cognitive capacities [141]. For example, older chimpanzees [142] and killer whales [143] associate with fewer partners than younger adults. It is possible that a similar process, although for different reasons, happens during development, when immatures narrow their association preferences from a variety of adult and immature partners to individuals of similar age and the same sex (see below). Second, the increase in immatures' grooming integration with age (contrary to the proximity network) may have two possible explanations. As they age, immatures could dedicate more time to grooming and less to play [47,144], allowing them to groom more frequently or a wider network of troop members. Concurrently, they may become more proficient groomers, thus becoming more desirable grooming partners [145].

The results on changes in partner preferences with age confirmed other studies’ findings [66,146]. Immatures groomed more with partners of similar age (both other immatures, ≤ 5 years old; and peers, ± 6 months age difference) while growing older. In addition, they both spent more time in proximity and groomed more with partners of the same sex as they aged, indicating strong social selectivity with age. Immatures could favour similar-age and same-sex partners over other group members because of their increasing independence from adult social partners and the benefit of forming (potentially) long-lasting bonds. In the first case, while adults are important sources of knowledge on what to eat [15], baboons are generally weaned and thus able to forage independently within the first year of life [16,46], which could allow greater social selectivity. In addition, while young immatures could benefit from the proximity of adult kin to receive agonistic support, this may become less important with age as older immatures have established their social rank [114]. Finally, juveniles may need less foraging time compared to adults (due to a relatively smaller body size), having thus more time to socialize with other immatures [136,143]. In the second case, long-lasting bonds are important because they can enhance fitness [5,6]. Bonding with individuals of similar age (e.g. paternal kin [147]) and the same sex could translate into stable social bonds [148–150]. Philopatric females could share a large part of their lives with similarly aged females [148], while males could disperse together at maturity [151] (see below).

Age changes in immature baboon social networks differed by sex, which had generally a strong effect in our models compared to the other variables (see electronic supplementary material, §6). The results of our study are consistent with males' gradual social peripheralization prior to dispersal in female-philopatric species [57,58]. In our first analyses (H1), we found that males formed relatively weaker bonds with their mothers and with maternal partners. First, the decrease in time spent with the mother was more pronounced for males than for females. Male primates’ physical distancing from their mothers could arise from the mothers directing more agonism towards sons than daughters [74] or males being more physically precocious than females (e.g. in play [63]). These patterns may lead males to spend time farther from the mother earlier than females. Nevertheless, males groomed proportionally more with their mothers than females. This pattern may derive from males' lower grooming integration, leading mothers to be important grooming partners for their sons. Rates for grooming given were qualitatively higher in females than in males, but both sexes received similar amounts of grooming (see electronic supplementary material, §8). If females receive grooming from more partners than males [146] (see below), they may have less time to dedicate to the mother. Second, males’ proximity and grooming networks resembled their mothers' less closely compared to females. In female-philopatric species, maternal kin form close bonds with each other [78,79]. These are more important for females compared to males for two reasons: maternal kin bonds will help females inherit and maintain their social rank (through agonistic support [152]), and they will last throughout their lives, potentially leading to fitness benefits [2,5]. Instead, when males reach a body size greater than that of adult females (at approx. 6 years old), they attain their social rank through agonistic interactions, without the support of their matriline [153]. Males also disperse at adulthood, leaving behind the social bonds formed in their natal troop [38], and thus can ‘afford’ to invest less in them during juvenescence.

Our second analyses (H2) showed that males became relatively less integrated in the grooming network as they aged. Males' relatively lower grooming network integration could derive from both their lower tendency to groom and their higher sexual segregation compared to females. In female-philopatric primate species, immature males groom less than females, but spend more time playing [58]. Play has been hypothesized to be important to acquire fighting skills for immature males, who will compete for dominance [135]. Focusing on same-sex grooming partners, males’ integration may diminish also because of lower availability of potential partners, while females share grooming more evenly with both sexes. Juvenile males may prefer to groom with each other because male bonding could lead them to emigrate together, increasing their probability of survival during dispersal and integration in a new group [151,154]. Despite males' gradual peripheralization, immatures’ grooming integration increased with age in both sexes. This is partly explained by the fact that infants of both sexes dedicate very little time to grooming, given that it is a behaviour learnt with time [144,145]. In addition, the increase in males' grooming activity may be explained by a similar increase in their sexual activity during development [155]: older juveniles may copulate with adult females at the early or late stages of their cycle (when fertility is low and females are not monopolized by a dominant male [156]) and exchange grooming with them [157,158].

The age and sex differences in network structure highlighted by our results may have important implications for social learning, and thus the formation of culture (i.e. behaviours shared by most members of a group and maintained through time [159]). In particular, we suggest that the emergence of culture, according to its most common definition (i.e. at the group level; but see [160]), in female-philopatric societies may be limited by age- and sex-driven social clustering [161]. Matrilines could be pivotal centres of information transmission for young immatures (e.g. in vervet monkeys [162,163]; in Japanese macaques *M. fuscata* [164,165]). However, the presence of social clusters around matrilines, as our results show, could diminish social information transmission across a group and instead cause it to cluster in matrilineal ‘subgroups’. This is because the individuals who tend to acquire information are socially separated from those who are more likely to produce it: (i) immatures, who rely more on social learning, and adults, who have a greater ability to produce information [15]; (ii) females, who learn more socially, and males, who learn more asocially [75,76]. Although these considerations remain speculative, this theoretical framework could help future studies to investigate the constraints leading to the formation of matrilineal ‘cultures’ as opposed to group cultures [161], ultimately questioning culture as a group-level behaviour [160].

## Data Availability

Research data and R scripts are available through the Dryad Digital Repository: Roatti, Vittoria; Cowlishaw, Guy; Huchard, Elise; Carter, Alecia (2023), Social network inheritance and differentiation in wild baboons, Dryad, Dataset, https://doi.org/10.5061/dryad.ffbg79cz1 [129]. The data are provided in the electronic supplementary material [166].
